# Bacteriology and the Medical Art

**Published:** 1892-08-13

**Authors:** 


### The Hospital

An Institutional Journal of

**Science, Medicine, Nursing, and Philanthropy.**

*For Hospitals, Asylums, Infirmaries, Dispensaries, Medical Practitioners, Students, Nurses, and the Charitable Public.*

Vol. XII.—No. 307.] [Aug. 13, 1892.

### Bacteriology and the Medical Art.

The modern medical man lives in a world of wonders. As he reads the stories of the destructions and ruins brought about by minute creatures which no unaided human eye can even so much as perceive, he involuntarily asks himself, How can these things be? It is not surprising that many medical men, competent practitioners too, refuse to believe that the gross and visible world is so largely at the mercy of the minute and practically invisible. Our fathers, say they, knew nothing of microbes, and yet they succeeded in curing their patients exceedingly well. That is no doubt so; and the aged practitioner who unhesitatingly trusts his past experience is a saner man than some of his youthful contemporaries who, microbe mad, cast the experience of former generations to the winds, and recklessly expose their suffering patients to the risks of methods that have nothing to recommend them but their newness. Quinine is still a tonic, microbe or no microbe; opium still continues to relieve pain, and blue pill to give comfort to the liver. To throw away the clinical experience of hundreds of careful and conscientious years would be the action of madmen, not of sane physicians.

But if he who locks a door on the past is a madman, so also is he who refuses to open his eyes to the present and to the future. The workers at bacteriological science have done their work so thoroughly, with so much patience, care, and endless repetition, that a reasonable mind is compelled to consider what they say, and to attach to it the full weight of their intelligent thoroughness. At the annual meeting of the British Medical Association the other day, Dr. Sims Woodhead made a gallant attempt to carry his science of bacteriology into the very centre and heart of daily medical practice. Let us, he said in effect, take the every-day subject of diphtheria. Now, there is hardly a man in the medical profession who has not had painful experiences with diphtheritic disease. Dr. Sims Woodhead gave his audience such an account of diphtheria that every intelligent man in the room could not but feel that there was in that account the possibility of such a victorious mastery of the disease as we had never felt confident of before. As is well known, there are some patients and diseases which give the doctor no anxiety whatever, because he knows that he can restore perfect health within a reasonable and definite time. But there are other diseases, and diphtheria is one of them, which put a constant strain upon the doctor's mind for weeks together. That strain bacteriology, it seems probable, is destined soon to relieve.

What is diphtheria? According to Dr. Sims Woodhead, it consists essentially of a bacterial colony or colonies situated and developing upon coagulated fibrin, on an inflamed and injured mucous membrane, which bacterial colony produces a poisonous secretion, the poisonous secretion in its turn reducing to a poisonous solution the coagulated fibrin or the cells of the inflamed mucous membrane. There is thus produced at the diseased locality a secondary fluid of a highly toxic character, a dialysable albumose, which can be as freely taken up by the blood and lymph vessels as any other rapidly dialysable fluid. It is this secondary product that seems to convert diphtheria from a local into a general disease; which product, acting upon the nerves and their terminations, produces the various diphtheritic paralyses, and which also, passing to every part of the body with the blood and lymph streams, results in fatty degeneration of the liver, the kidneys, and other organs, the preservation of whose structural integrity is so fundamental to life.

If this be a true account of diphtheria, and it certainly commends itself, step by step, to the instructed physiologist, it is easy to see exactly where the physician should come in with his remedies to secure a favourable result with practical uniformity and certainty. Any medical man who has followed the reasoning in detail will see at once how very easy it may be to cure diphtheria at the very first stage of its progress, and how very difficult at the final stage. At first we have a disease strictly limited to a mere patch of the throat; at the last we have the whole of the blood and the lymph of the body charged with destructive poison, and that poison in full operation in the muscles, the nerves, the secreting and the excreting organs, reducing their cell elements to still further poisonous products, and so carrying the vital citadel by what often proves an assault altogether overwhelming. What can we do when the disease has reached this terribly deadly stage? To what element in the bodily structure shall we make our therapeutic appeal? The blood is charged with poison; the lymph, too, is charged; and the very cells of every organ and tissue in the body are, as it were, throwing down their arms and deserting *en masse* to the enemy.

It is plain that if diphtheria is to be dealt with in the way that is most intelligent, the primary and secondary poisons produced by the bacilli lodged in the throat must be rigorously excluded from the blood and lymph. In other words, diphtheria must not be allowed to get beyond the stage of a purely local disease. General experience shows us that it is seldom the local bacterial colonies which kill, but the primary and secondary poisons originated by those colonies. The problem then is to diagnose the disease before the bacteria have yielded up their poisonous secretion, or at the latest before that primary poisonous secretion has converted the coagulated fibrin lying upon the mucous membrane into a secondary poisonous product equally active and equally deadly. Is there any certain method of diagnosing diphtheria, with its present bacilli, at a stage antecedent to the production of their poisonous secretion? Dr. Sims Woodhead thinks there is. If every medical man were a practical bacteriologist the thing, it is obvious, would be easy. Dr. Woodhead gives a method whereby every intelligent practitioner may become a practical bacteriologist in so far as diphtheria is concerned. Readers who are in medical practice will do well to consult Dr. Woodhead's paper on this point.

In the meantime, however, let it be pointed out that in a large number of cases it is possible not only to confine the attack of diphtheria to the throat and to prevent it altogether from becoming a general disease, but even to begin a stage earlier and to anticipate the bacterial incursion, and so to help our patients to avoid the deadly seizure altogether. The diphtheria bacillus, Dr. Woodhead informs us, does not readily attack the healthy throat. Even simple sore throats, where there is no exudation of coagulating fibrin, do not offer a very encouraging nidus to the bacillus. But if an inflammatory throat take to exuding coagulable fibrin rapidly, or if it be wounded or injured in any way, then the diphtheria bacillus finds exactly the conditions it most appreciates for its rapid and dangerous developments.

Now, there is a very obvious moral here, and a moral of the utmost practical importance. Given an inflamed throat which is exuding fibrin, we have to destroy the coagulated fibrin as rapidly as possible, and to reduce the inflammation in the mucous membrane so that exudation may promptly cease. This is hardly the place for entering upon a consideration of methods. But there are two proceedings, which really resolve themselves into one, and which if faithfully carried out would in all probability reduce diphtheria to a surprising minimum. The use of mercury, locally and internally, is the double barrelled method here recommended. A full dose of calomel given internally, will in a few hours abate the local inflammation, and lower the temperature in quite a remarkable degree, and the frequent gentle swabbing of the throat with a weak solution of mercuric iodide will render the coagulated fibrin harmless, and destroy any scattered bacteria which may be beginning to congregate and settle down to their deadly work.

Is this reasoning sound? We think it is. If it be then, in diphtheria, at any rate, a distinct advance in medical science is made all along the line. Be that as it may, we feel that medical practitioners as a class ought to accept Dr. Sims Woodhead's admirably reasoned conclusions as the bases of prolonged experimentation. Diphtheria and its management are, like many other diseases and their treatment, still to a large extent in a state of chaotic darkness. We can accept Dr. Woodhead's conclusions without any risk to our patients. On the contrary, the early diagnosis of diphtheria and its prompt treatment by bactericides are entirely to the good. Still more is it to the patient's advantage if we can anticipate the bacterial onslaught, and by agents which reduce inflammation and destroy local sepsis, preserve him altogether from the scourge of a dangerous disease. The medical profession is grateful to men like Dr. Woodhead for their practical work and their intelligent teaching. How much more grateful would the public be if it were but educated enough to know light from darkness in these vital matters!

